# Risk factors associated with cancer and metabolic encephalopathy in Alzheimer’s disease patients

**DOI:** 10.3389/fnagi.2026.1810937

**Published:** 2026-05-26

**Authors:** Danny Pham, Connor O’Brien, Jillian Florez-Bhandari, Nathan Faulstich, Timi Ojo, Sarah Aloi, Richard Goodwin, Laurie Roley, Samuel I. Nathaniel, Thomas I. Nathaniel

**Affiliations:** 1 University of South Carolina School of Medicine Greenville, Greenville, SC, United States; 2 Biomedical Engineering Molinaroli College of Engineering and Computing, North Greenville University, Columbia, SC, United States; 3 PRISMA Health Greenville, Greenville, SC, United States

**Keywords:** Alzheimer disease, cancer, dementia, metabolic encephalopathy, risk factors

## Abstract

**Background:**

Alzheimer’s disease (AD) frequently coexists with risk factors that modify its clinical course. The combined presence of cancer and metabolic encephalopathy (ME) in AD represents a particularly vulnerable and understudied phenotype. We investigated whether cancer-associated risk profiles differ between AD patients with and without metabolic encephalopathy.

**Methods:**

We used multivariate logistic regression to identify clinical, vascular, pulmonary, neurocognitive, psychiatric, and treatment-related factors distinguishing (i) AD patients with metabolic encephalopathy with and without cancer (AD + ME ± C) and (ii) AD patients without metabolic encephalopathy with and without cancer (AD – ME ± C). Adjusted odds ratios (ORs) with 95% confidence intervals (CIs) were used to identify risk factors and phenotype-specific associations.

**Results:**

Of the total cohort, 10,516 patients had metabolic encephalopathy, and 118,253 did not. Cancer coexistence was present in 146 AD + ME patients and 1,167 AD – ME patients. Among AD + ME patients, cancer was strongly associated with cerebrovascular accident (OR = 3.47, 95% CI 2.16–5.59), secondary dementia (OR = 9.89, 95% CI 3.26–29.98), mild cognitive impairment (OR = 5.20, 95% CI 1.98–13.27), chronic obstructive pulmonary disease (OR = 7.66, 95% CI 5.20–11.29), and SSRI use (OR = 3.27, 95% CI 2.21–4.87). In contrast, memantine, buspirone, and valproate were associated with AD + ME without cancer. Among AD–ME patients, cancer was associated with dyslipidemia, peripheral vascular disease, congestive heart failure, arteriosclerosis, COPD, and cutaneous ulcers, reflecting chronic systemic illness.

**Conclusion:**

Metabolic encephalopathy was associated with a different clinical profile in cancer-associated AD. Patients with ME exhibited increased systemic and neurologic vulnerability (e.g., vascular comorbidity and frailty indicators) rather than differences in baseline cognitive severity alone.

## 1 Introduction

Alzheimer’s disease (AD) is a progressive neurodegenerative disorder characterized by synaptic dysfunction, neuronal loss, and cumulative cognitive decline (Tenchov et al., 2024). With global population aging, an increasing proportion of patients with AD present with complex multimorbidity, including systemic malignancies and metabolic disturbances that substantially worsen prognosis and complicate clinical management (Stirland et al., 2025). Among these, the coexistence of cancer (C) and metabolic encephalopathy (ME) defines a particularly vulnerable clinical state, marked by acute cognitive decompensation, high rates of delirium, limited treatment tolerance, and increased clinical complexity (Majd et al., 2019).

Epidemiologic studies consistently describe an inverse association between AD and cancer incidence (Majd et al., 2019). Cancer survivors demonstrate an approximately 30–35% reduced risk of subsequent AD, while individuals with AD exhibit a 35–50% lower incidence of several malignancies, including lung, colorectal, prostate, and bladder cancers (Musicco et al., 2013; Ma et al., 2025; Driver et al., 2012). This paradox has been attributed to opposing biological processes, including dysregulated cell-cycle re-entry and apoptosis in AD versus enhanced cellular survival and proliferation in cancer, as well as shared molecular pathways involving p53, PIN1, Wnt signaling, oxidative stress responses, and mitochondrial dysfunction (Behrens et al., 2009; Aylon and Oren, 2016). In addition, exposure to certain cancer therapies such as chemotherapy and endocrine agents, including tamoxifen, has been associated with a reduced long-term risk of AD, potentially through modulation of estrogen signaling, inflammation, and amyloid processing (Frain et al., 2017).

Despite this inverse relationship at the population level, clinical outcomes are markedly worse when AD and cancer coexist in the same patient (Zabłocka et al., 2021). Alzheimer’s disease is associated with delayed cancer diagnosis, restricted access to curative therapies, increased treatment-related toxicity, and substantially worse clinical outcomes, as reported in a prior study (Romero et al., 2014). Prior studies report that approximately one-third of AD patients die within 6 months of a cancer diagnosis, compared with fewer than 10% of cognitively intact patients, reflecting both disease severity and challenges in oncologic decision-making (Caba et al., 2021; Raji et al., 2008; Mezencev and Chernoff, 2020).

ME is associated with increased clinical risk in this population and represents one of the most common and potentially reversible causes of acute cognitive deterioration (Ojo et al., 2025). ME encompasses a spectrum of diffuse cerebral dysfunction resulting from systemic metabolic derangements, including electrolyte abnormalities, hepatic or renal failure, hypoxia, infection, endocrine disturbances, and medication toxicity (Angel et al., 2008). In oncology populations, ME is a leading cause of altered mental status and delirium, frequently precipitated by chemotherapy, opioids, corticosteroids, immune checkpoint inhibitors, or tumor-related organ dysfunction (Breitbart and Alici, 2012; Inouye et al., 2014). In patients with AD, toxic–metabolic encephalopathy can acutely exacerbate baseline cognitive impairment, accelerate functional decline, and precipitate prolonged hospitalization (Mathews et al., 2014).

Clinically, ME is distinguished from AD by its rapid onset (hours to days), fluctuating course, and impaired level of consciousness (Martins and Fernandes, 2012); however, the two frequently coexist and are difficult to disentangle in advanced dementia. Importantly, up to 10% of individuals diagnosed with AD may have unrecognized cirrhosis and hepatic encephalopathy, underscoring the potential for misdiagnosis and missed therapeutic opportunities (Silvey et al., 2024; Vilstrup et al., 2014). AD further increases susceptibility to delirium and metabolic decompensation through reduced cognitive reserve, impaired neurovascular coupling, chronic neuroinflammation, and blood–brain barrier dysfunction (Chen et al., 2023).

Cancer therapies compound this vulnerability. Cytotoxic chemotherapy, radiation therapy, and targeted agents can induce electrolyte disturbances, mitochondrial dysfunction, and systemic inflammation, thereby increasing the risk of ME and delirium (Gorini et al., 2018). Conversely, cognitive impairment may limit adherence to cancer treatment and heighten vulnerability to drug–drug interactions that exacerbate metabolic instability (Aktepe and Aktepe, 2026). Sex-specific differences have also been reported, with female AD patients demonstrating higher rates of coexisting cancer and additional comorbidities such as osteoporosis and infection, suggesting hormonal, immunologic, or care-access influences (Sundbøll et al., 2018).

Risk factors contributing to the overlap of AD, ME, and cancer (AD + ME + C) can be conceptualized as baseline vulnerability (predisposing factors) and acute triggers (precipitating factors) (Kodam et al., 2023). Predisposing factors include dementia severity, advanced age, frailty, functional impairment, polypharmacy, and cardiovascular or cerebrovascular disease burden, all of which reduce cerebral metabolic resilience and increase delirium risk (Mei et al., 2023). Hepatic vulnerability is particularly important, as a subset of cognitively impaired patients may harbor unrecognized cirrhosis with potentially treatable hepatic encephalopathy (Bajaj et al., 2024). Precipitating risk factors are frequently encountered during cancer care and include metabolic derangements related to renal or hepatic dysfunction, dehydration, electrolyte imbalance, infection, hypoxia, and medication toxicity, especially opioids and sedative agents whose effects are amplified in older adults with AD (Byar and Anderson, 2025).

Although the coexistence of AD and cancer is associated with poor clinical outcomes associated with multiple interacting risk factors (You et al., 2026), the incremental contribution of metabolic encephalopathy to this risk remains incompletely characterized. Moreover, the specific risk factors underlying this added complexity are yet to be systematically investigated. Therefore, it is possible that both predisposing and precipitating risk factors are not uniformly distributed between AD patients with cancer who develop ME (AD + ME + C) and AD with cancer but without metabolic encephalopathy (AD – ME + C). Accordingly, the first objective of this study is to compare risk factors between the AD + ME + C and AD – ME+C groups and to determine whether these risk factors differ between them.

The coexistence of AD with cancer and metabolic encephalopathy is associated with a distinct clinical profile and greater vulnerability compared with Alzheimer’s disease patients with cancer without metabolic encephalopathy. While AD patients with cancer but without metabolic encephalopathy may experience gradual cognitive decline consistent with their underlying neurodegenerative disease, the superimposition of metabolic encephalopathy is associated with abrupt, fluctuating deterioration in mental status, increased delirium burden, impaired treatment tolerance, and substantially worse clinical outcomes. This distinction underscores the likelihood that Alzheimer’s disease with cancer and metabolic encephalopathy and Alzheimer’s disease with cancer without metabolic encephalopathy are associated with different risk profiles. Therefore, the second objective is to assess risk factors associated with (i) AD patients with metabolic encephalopathy with and without cancer (AD + ME ± C), and (ii) AD patients without metabolic encephalopathy with and without cancer (AD – ME ± C) using prospective registry data from patients admitted to a primary Alzheimer’s disease center between 2010 and 2013.

Investigating factors associated with AD patients with and without ME, stratified by cancer status, is essential for understanding the heterogeneity of risk factors associated with clinical trajectories in this high-risk population. ME represents an acute, potentially reversible contributor to cognitive deterioration that markedly worsens outcomes when superimposed on AD, particularly in patients with cancer, where it is associated with delirium and treatment intolerance, as well as adverse clinical outcomes. Identifying risk factors in AD patients with ME can enable earlier recognition and targeted metabolic correction, whereas examining factors in AD patients without ME provides a critical baseline for distinguishing chronic disease burden from acute metabolic triggers. Together, these complementary analyses help delineate distinct AD phenotypes, identify patients at risk of transition to metabolic decompensation, and inform risk-adapted, preventive, and patient-centered management strategies as the prevalence of AD and cancer continues to rise.

## 2 Methods

### 2.1 Study population

We utilized 5-year data from the Prisma Health Alzheimer’s Dementia (AD) registry. The protocol of this study was approved by the Institutional Review Board and the Prisma Health Institutional Committee for Ethics. Patients were classified as having Alzheimer’s disease (AD) based on standardized clinical diagnostic criteria consistent with the National Institute on Aging–Alzheimer’s Association (NIA-AA) framework. Diagnosis incorporated a combination of validated cognitive assessments (e.g., Mini-Mental State Examination [MMSE], Montreal Cognitive Assessment [MoCA]), structural neuroimaging (MRI/CT) to exclude alternative etiologies, and laboratory testing to rule out reversible causes of cognitive impairment. This multimodal approach reflects contemporary clinical practice in tertiary care dementia centers.

### 2.2 Data collection

Data were collected from electronic medical records of patients that present several risk factors, including hypertension (defined as blood pressure greater than 140/90), dyslipidemia (abnormal lipid or triglyceride levels in the blood), and peripheral vascular disease (a condition characterized by narrowing or blockage of blood vessels outside the brain and heart). Data were also collected on AD patients with metabolic encephalopathy diagnosed based on neuroimaging and laboratory testing. This analysis also included other factors, such as vitamins, medications, and specific pathologies documented in patients’ charts.

Data were also collected on demographics, comorbidity, laboratory values, and medication. Patient demographics included age, race, and ethnicity. We collected data on several risk factors including insomnia, cerebrovascular accidents, osteoporosis, gait dysfunction, atrial fibrillation, hallucination, cancer, anxiety, urinary tract infection, upper respiratory tract infections, thyroid disease, insulin use, secondary dementia, cold sore, frailty, GI ulceration, lung adenocarcinoma, small cell carcinoma of the lung, headache, congestive heart failure, obstructive sleep apnea, arteriosclerosis, cutaneous ulcers, psychosis, COPD, traumatic head injury, vasospasm, squamous cell carcinoma of the lung, pneumonia, large cell carcinoma of the lung, rheumatoid arthritis, ductal carcinoma of the breast, subclavian artery disease, microangiopathy, mild cognitive impairment, teratoma, microangiopathy, down syndrome, gastric erosion, senile tremor, and alcohol use. Medication history included valproate, buspirone, memantine, second-generation antipsychotics (SGAP), Carbonic anhydrase inhibitors, and selective serotonin reuptake inhibitors. Data were collected on laboratory results, including folate, vitamin D, TSH, magnesium, serum calcium, and homocysteine levels.

Metabolic encephalopathy (ME) was defined as a clinically documented toxic–metabolic encephalopathy characterized by acute or subacute alterations in mental status attributable to systemic metabolic or physiologic disturbances. Metabolic encephalopathy cases were identified using ICD-based coding in the electronic medical record, including codes such as ICD-10 G93.41 (metabolic encephalopathy), G93.49 (other encephalopathy), and related codes indicative of toxic–metabolic etiologies. These codes were used in conjunction with physician documentation and supporting laboratory findings to improve diagnostic specificity. To improve diagnostic specificity, ME classification required the presence of an identifiable metabolic or systemic precipitant, distinguishing it from primary psychiatric conditions or nonspecific delirium without a clear metabolic etiology. While delirium and metabolic encephalopathy share overlapping clinical features, only cases with documented metabolic or toxic causes were included under ME. The registry predominantly captures acute or subacute encephalopathic events rather than chronic cognitive impairment; therefore, baseline neurodegenerative decline due to Alzheimer’s disease was not classified as ME. Hepatic encephalopathy was included as a subtype of metabolic encephalopathy when documented in association with liver dysfunction or cirrhosis. Neuroimaging and laboratory testing were used in clinical practice to exclude structural or alternative causes of altered mental status and to support the diagnosis of metabolic encephalopathy.

### 2.3 Statistical analysis

All statistical analyses were conducted using IBM SPSS Statistics for Mac, version 30.0.0.0 (IBM Corp., Armonk, NY). Univariate analyses were performed on the full cohort of patients with AD to identify statistical differences between patients with and without metabolic encephalopathy. Continuous variables with a normal distribution were presented as mean ± SD and were assessed for significance using a Student *T*-Test. Categorical variables were reported as frequencies and analyzed using Pearson’s chi-squared test. The cohort was further stratified by cancer status. Within each subgroup, univariate analyses were conducted to determine cancer-specific differences in clinical risk factors and comorbidities.

Multivariable logistic regression models were constructed to identify factors independently associated with each outcome of interest. Candidate variables were selected based on clinical relevance and results from univariate analyses, with variables demonstrating statistical significance or near-significance (*p* < 0.30) considered for inclusion to avoid exclusion of potentially important predictors.

A backward stepwise selection approach based on the likelihood ratio test was applied to derive the final models. Variables were sequentially removed based on their contribution to model fit, while ensuring retention of clinically meaningful covariates.

To address potential confounding, models included variables spanning multiple domains, including demographic characteristics, cardiovascular comorbidities, cancer-related variables, neuropsychiatric conditions, and medication use. This approach was intended to estimate independent associations while minimizing omitted variable bias.

Multicollinearity was assessed using variance inflation factors (VIFs) and tolerance statistics. A VIF threshold > 5 and tolerance < 0.2 were used to identify problematic collinearity. No variables in the final models exceeded these thresholds. Where clinically related variables demonstrated conceptual overlap (e.g., vascular comorbidities or cancer subtypes), their inclusion was evaluated based on contribution to model stability and goodness-of-fit.

Model performance was evaluated using discrimination (area under the receiver operating characteristic curve, AUROC) and calibration (Hosmer–Lemeshow test). Given the large sample size, the Hosmer–Lemeshow test was interpreted cautiously, as it is highly sensitive and may indicate statistically significant deviations from perfect calibration even when miscalibration is minimal.

Statistical significance was defined as a *p*-value less than 0.05. Odds ratios (ORs) for clinical risk factors and comorbidities were reported with 95% confidence intervals (CIs). Model performance metrics, including classification accuracy and AUROC, were calculated on the same dataset used for model fitting (apparent performance). No internal validation procedures (e.g., bootstrapping or cross-validation) were applied. The ratio of events to predictor variables (events per variable, EPV) was considered to assess potential overfitting. While the overall cohort size was large, subgroup analyses with fewer outcome events may reduce EPV, increasing the risk of model instability. Variables with low prevalence or sparse event counts were interpreted cautiously to avoid model instability and inflated effect estimates.

## 3 Results

### 3.1 Baseline demographic and risk factors

**Figure 1:**
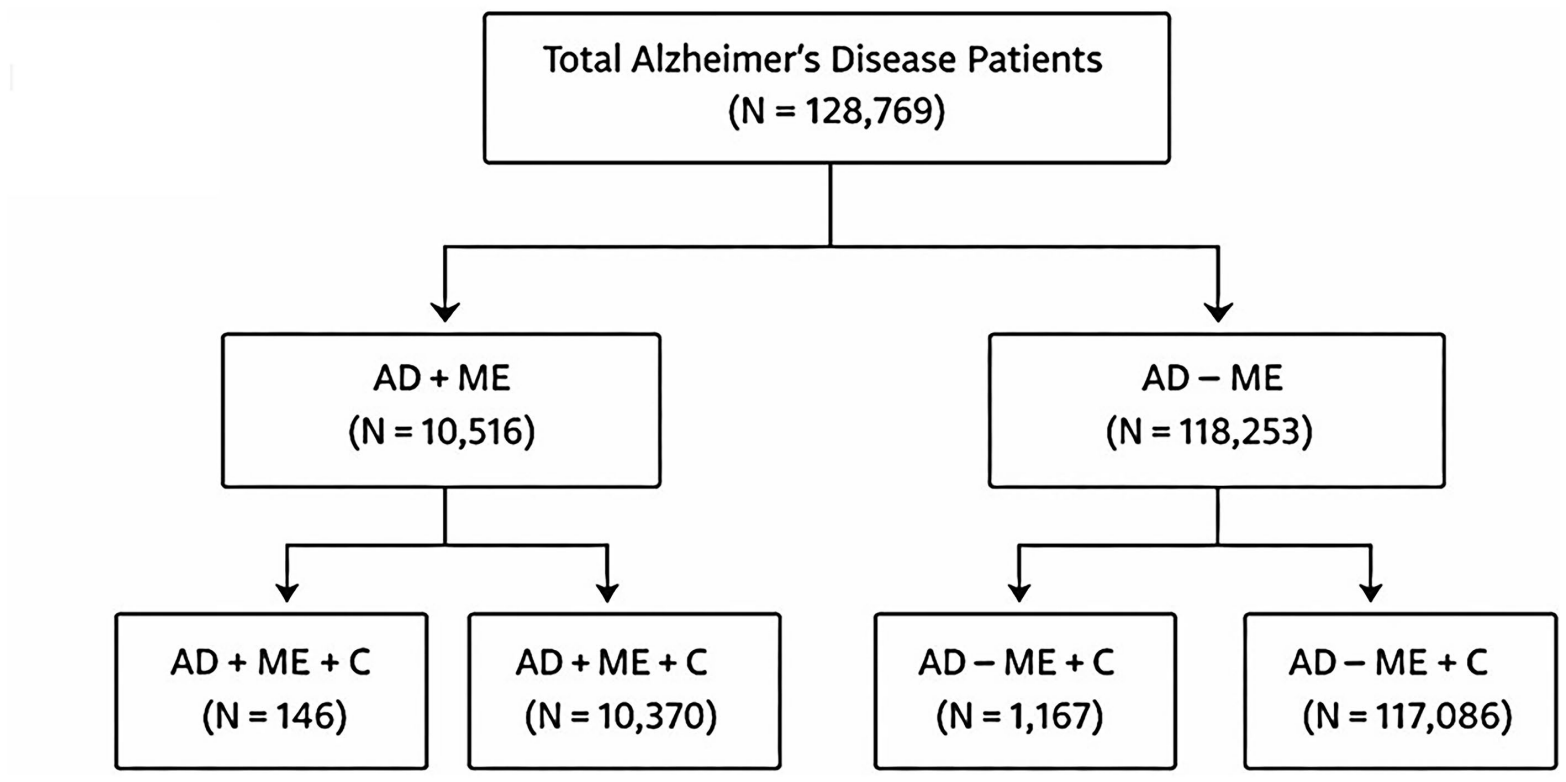
The stepwise derivation of the analytic cohorts from the initial 128,769 Alzheimer’s disease (AD) patients in the registry. The diagram delineates stratification by metabolic encephalopathy (ME) status and subsequent classification by cancer status, resulting in the four analytic groups.

A total of 128,769 patients diagnosed with AD were identified (Table 1). Figure 1 illustrates the stepwise derivation of the analytic cohorts from the initial 128,769 Alzheimer’s disease (AD) patients in the registry. The diagram delineates stratification by metabolic encephalopathy (ME) status and subsequent classification by cancer status, resulting in the four analytic groups: AD + ME + C, AD + ME − C, AD – ME + C, and AD−ME−C. Among them, 10,516 presented with metabolic encephalopathy, while 118,253 did not. AD patients with metabolic encephalopathy were significantly older (*p* < 0.001). Those without metabolic encephalopathy were more likely to be Hispanic (*p* < 0.002). Patients in the metabolic encephalopathy group had higher rates of atrial fibrillation, cancer, urinary tract infections, frailty, advanced age, congestive heart failure, chronic obstructive pulmonary disease (COPD), traumatic brain injury, squamous cell carcinoma of the lung, pneumonia, rheumatoid arthritis, and gastric erosion (all *p* < 0.05). In contrast, non-metabolic encephalopathy patients were more likely to have insomnia, dyslipidemia, cerebrovascular accidents, osteoporosis, anxiety, upper respiratory infections, secondary dementia, cold sores, mild cognitive impairment, headache, obstructive sleep apnea, and Down syndrome (all *p* < 0.05). Regarding treatment, patients with metabolic encephalopathy were more likely to receive buspirone (*p* < 0.001), while those without metabolic encephalopathy were more frequently treated with selective serotonin reuptake inhibitors (SSRIs), carbonic anhydrase inhibitors, and memantine (all *p* < 0.001). Additionally, patients with non-metabolic encephalopathy had significantly higher serum magnesium and calcium levels (p < 0.001).

**Table 1:** Demographic and clinical characteristics of Alzheimer’s patients stratified by the diagnosis of metabolic encephalopathy.

Characteristic	Without encephalopathy	With encephalopathy	*P-*value
Number of patients	118,253	10,516	
Gender: No. (%)			
Male	51,116 (43.2)	4,757 (45.2)	<0.001*^a^
Female	67,137 (56.8)	5,759 (54.8)	
Age Group: No. (%)			
<50	19,140 (16.2)	1,178 (11.2)	<0.001*^a^
50–59	12,171 (10.3)	1,432 (13.6)	
60–69	19,213 (16.2)	1951 (18.6)	
70–79	26,517 (22.4)	2,844 (27.0)	
80–89	26,911 (22.8)	2016 (19.2)	
90+	14,301 (12.1)	1,095 (10.4)	
Mean ± SD	68.5 **±** 20.6	70.0 **±** 15.9	<0.001*^b^
Race: No (%)			
White	94,262 (79.7)	8,840 (80.3)	<0.001*^a^
Black	19,072 (16.1)	1702 (16.2)	
Other	585 (0.5)	30 (0.3)	
Hispanic Ethnicity: No. (%)	2,104 (1.8)	101 (1.0)	
Medical History: No. (%)			
Hypertension	62,484 (52.8)	5,552 (52.8)	0.932^a^
Insomnia	8,256 (7)	650 (6.2)	0.002*^a^
Dyslipidemia	30,390 (25.70)	2,209 (21.0)	<0.001*^a^
Cerebrovascular accidents	12,563 (10.6)	958 (9.1)	<0.001*^a^
Osteoporosis	11,269 (9.5)	915 (8.7)	0.005*^a^
Gait dysfunction	4,283 (3.6)	234 (2.2)	<0.001*^a^
Peripheral vascular disease	7,870 (6.7)	785 (7.5)	0.001*^a^
Atrial fibrillation	9,460 (8.0)	978 (9.3)	<0.001*^a^
Hallucination	1,133 (1.0)	84 (0.8)	0.106^a^
Anxiety	12,806 (10.8)	980 (9.3)	<0.001*^a^
Cancerunspecified	1,167 (1)	146 (1.4)	<0.001*^a^
UTI	13,124 (11.1)	1,565 (14.9)	<0.001*^a^
Upper respiratory infection	2,658 (2.2)	147 (1.4)	<0.001*^a^
Thyroid disease	624 (0.5)	59 (0.6)	0.652^a^
Insulin use	1,088 (0.9)	79 (0.8)	0.08^a^
Secondary dementia	2,323 (2)	83 (0.8)	<0.001*^a^
Cold sore	324 (0.3)	2 (0.0)	<0.001*^a^
Frailty	150 (0.1)	28 (0.3)	<0.001*^a^
GI ulceration	878 (0.7)	72 (0.7)	0.507^a^
Lung adenocarcinoma	192 (0.2)	18 (0.2)	0.830^a^
Small cell carcinoma of the lung	181 (0.2)	20 (0.2)	0.355^a^
Advanced age	2082 (1.8)	246 (2.3)	<0.001*^a^
Mild cognitive impairment	6,650 (5.6)	101 (1.0)	<0.001*^a^
Headache	2,192 (1.9)	142 (1.4)	<0.001*^a^
Congestive heart failure	7,485 (6.3)	1,159 (11.0)	<0.001*^a^
Obstructive sleep apnea	12,337 (10.4)	946 (9.0)	<0.001*^a^
Arteriosclerosis	328 (0.3)	13 (0.1)	0.003*^a^
Cutaneous ulcer	211 (0.2)	30 (0.3)	0.015^a^
Psychosis	946 (0.8)	44 (0.4)	<0.001*^a^
COPD	12,475 (10.5)	1,311 (12.5)	<0.001*^a^
Traumatic head injury	628 (0.5)	101 (1.0)	<0.001*^a^
Vasospasm	9 (0.0)	0 (0.0)	0.371^a^
Squamous cell carcinoma of the lung	63 (0.1)	33 (0.3)	<0.001*^a^
Pneumonia	14,009 (11.8)	2,181 (20.7)	<0.001*^a^
Large cell carcinoma of the lung	3 (0.0)	0 (0.0)	0.605^a^
Rheumatoid arthritis	1,515 (1.3)	179 (1.7)	<0.001*^a^
Ductal carcinoma of the breast	7 (0.0)	0 (0.0)	0.430^a^
Subclavian artery disease	15 (0.0)	0 (0.0)	0.248^a^
Teratoma	5 (0.0)	0 (0.0)	0.505^a^
Microangiopathy	2 (0.0)	0 (0.0)	0.673^a^
Down syndrome	152 (0.1)	1 (0.0)	<0.001*^a^
Gastric erosion	4 (0.0)	9 (0.1)	<0.001*^a^
Senile tremor	10 (0.0)	0 (0.0)	0.346
Hispanic	2,411 (2.0)	169 (1.6)	0.002*^a^
Alcohol use	32,241 (28.1)	3,017 (28.8)	0.092^a^
Medication use No. (%)			
SSRI -Present	37,658 (31.8)	3,163 (30.10)	<0.001*^a^
CAI-Present	29,105 (24.6)	1,012 (9.6)	<0.001*^a^
SGAP Present	18,921 (16.0)	1715 (16.3)	0.409^a^
Memantine	19,084 (16.1)	699 (6.6)	<0.001*^a^
Buspirone	10,990 (9.3)	1,187 (11.3)	<0.001*^a^
Valproate	18,037 (15.3)	1,502 (14.3)	0.008^a^
Lab values: Mean ± SD			
Folate	11.7 **±** 4.2	11.3 **±** 4.2	0.018*^b^
B12	820.4 **±** 383.9	-	0.239^b^
Vitamin D	28.9 **±** 15.3	29.8 **±** 15.3	0.431^b^
TSH	3.2 **±** 10.6	3.4 **±** 10.2	0.221^b^
Magnesium	2.0 **±** 0.3	1.9 **±** 0.4	<0.001*^b^
Serum calcium	9.1 **±** 0.7	9.0 **±** 0.8	<0.001*^b^
Homocysteine	14.9 **±** 13.0	12.9 **±** 5.0	0.500^b^

Results for continuous variables are presented as Mean ± SD, while discrete data are presented as percentage frequency. Pearson’s Chi-Square test is used to compare clinical risk factors and demographic characteristics between patients with and without metabolic encephalopathy. ^a^Pearson’s Chi-Squared test. ^b^Student’s *T-*test. *P-value < 0.05.

Table 2 summarizes the demographics, clinical risk factors, medications, comorbidities, and laboratory results of patients with AD, stratified by the presence or absence of metabolic encephalopathy and cancer (AD±ME±C). AD patients with both metabolic encephalopathy and cancer were significantly older (*p* < 0.001) compared to those without metabolic encephalopathy. Among patients without metabolic encephalopathy and without cancer (AD−ME−C), there were higher rates of insomnia, gait dysfunction, urinary and respiratory tract infections, insulin use, secondary dementia, gastrointestinal ulceration, advanced age, mild cognitive impairment, and psychosis (all *p* < 0.05). Non-encephalopathic patients with cancer were more likely to present with hypertension, dyslipidemia, peripheral vascular disease, atrial fibrillation, anxiety, lung adenocarcinoma, small cell carcinoma of the lung, congestive heart failure, obstructive sleep apnea, arteriosclerosis, cutaneous ulcers, COPD, squamous cell carcinoma of the lung, pneumonia, large cell carcinoma of the lung, and ductal carcinoma of the breast (all *p* < 0.05). Among non-encephalopathic patients without cancer, those treated with selective serotonin reuptake inhibitors (SSRIs) and second-generation antipsychotics (SGAPs) were more likely to receive these agents (*p* < 0.001), and they also had higher magnesium levels (*p* = 0.03).

**Table 2:** Demographic and clinical characteristics of Alzheimer’s patients with metabolic encephalopathy and Alzheimer’s patients without metabolic encephalopathy stratified by cancer.

Characteristic	Without metabolic encephalopathy			With Metabolic encephalopathy		
	Without cancer	With cancer	*P-*value	Without cancer	With cancer	*P-*value
Number of patients	116,086	1,167		10,370	146	
Age group: No. (%)						
<50	19,113 (16.3)	27 (2.3)	<0.001*^a^	1,178 (11.4)	0 (0)	<0.001*^a^
50–59	12,060 (10.3)	111 (9.5)		1,399 (13.5)	33 (22.6)	
60–69	18,992 (16.2)	291 (24.9)		1905 (18.4)	46 (31.5)	
70–79	26,210 (22.4)	307 (26.3)		2,808 (27.1)	36 (24.7)	
80–89	26,567 (22.7)	344 (29.5)		1989 (19.2)	27 (18.5)	
90+	14,214 (12.1)	87 (7.5)		1,091 (10.5)	4 (2.7)	
Mean ± SD	68.4 **±** 20.7	73.8 **±** 11.8	<0.001*^b^	70.0 **±** 16.0	69.4 **±** 9.8	<0.001*^b^
Race: No (%)						
White	93,344 (79.7)	918 (78.7)	<0.001*^a^	8,313 (80.16)	127 (86.98)	0.534^a^
Black	18,853 (16.1)	219 (18.8)		1,683 (16.23)	19 (13.01)	
Other	564 (0.5)	21 (1.8)		30 (0.3)	0 (0.0)	
Hispanic Ethnicity: No. (%)	2,104 (1.8)	0 (0)		101 (1.0)	0 (0)	
Medical History: No. (%)						
Hypertension	61,755 (52.7)	729 (62.5)	<0.001*^a^	5,337 (52.5)	105 (71.9)	<0.001*^a^
Insomnia	8,206 (7)	50(4.4)	<0.001*^a^	633 (6.1)	17 (11.6)	0.006*^a^
Dyslipidemia	29,929 (25.6)	461 (39.5)	<0.001*^a^	2,177 (21)	32 (21.9)	0.785^a^
Cerebrovascular accidents	12,428 (10.6)	135 (11.6)	0.293^a^	933 (9)	25 (17.1)	<0.001*^a^
Osteoporosis	11,168 (9.5)	101 (8.7)	0.306^a^	892 (8.6)	23 (15.8)	0.002*^a^
Gait Dysfunction	4,254 (3.6)	29 (2.5)	0.037*^a^	229 (2.2)	5 (3.4)	0.322^a^
Peripheral vascular disease	7,712 (6.6)	158 (13.5)	<0.001*^a^	772 (7.4)	13 (8.9)	0.505^a^
Atrial fibrillation	9,344 (8.0)	116 (9.9)	0.014*^a^	970 (9.4)	8 (5.5)	0.109^a^
Hallucination	1,126 (1.0)	7 (0.6)	0.207^a^	84 (0.8)	0 (0)	0.275^a^
Anxiety	12,652 (10.8)	154 (13.2)	0.009*Dy^a^	974 (9.4)	6 (4.1)	0.029*^a^
UTI	13,038 (11.1)	86 (7.4)	<0.001*^a^	1,552 (15)	13 (8.9)	0.041^*a^
URI	2,647 (2.3)	11 (0.9)	0.003*^a^	147 (1.4)	0 (0)	0.147^a^
Thyroid disease	622 (0.5)	2 (0.2)	0.091^a^	58 (0.6)	1 (0.7)	0.840^a^
Insulin use	1,084 (0.9)	4 (0.3)	0.038*^a^	79 (0.8)	0 (0)	0.290^a^
Secondary Dementia	2,316 (2.0)	7 (0.6)	<0.001*^a^	79 (0.8)	4 (2.7)	0.028*^a^
Cold sore	324 (0.0)	0 (0)	0.072^a^	2 (0)	0 (0)	0.867^a^
Frailty	150 (0.1)	0 (0.0)	0.221^a^	28 (0.3)	0 (0)	0.530^a^
GI ulceration	878 (0.7)	0 (0.0)	0.003*^a^	72 (0.7)	0 (0)	0.312^a^
Lung adenocarcinoma	0 (0)	192 (16.5)	<0.001*^a^	0 (0)	18 (12.3)	<0.001*^a^
Small cell carcinoma of the lung	0 (0)	181 (15.5)	<0.001*^a^	0 (0)	20 (13.7)	<0.001*^a^
Advanced age	2078 (1.8)	4 (0.3)	<0.001*^a^	246 (2.4)	0 (0)	0.062^a^
Mild cognitive impairment	6,603 (5.6)	47 (4.0)	0.017*^a^	96 (0.9)	5 (3.4)	0.002*^a^
Headache	2,179 (1.9)	13 (1.1)	0.06^a^	142 (1.4)	0 (0.0)	0.155^a^
Congestive heart failure	7,342 (6.3)	143 (12.3)	<0.001*^a^	1,142 (11.0)	17 (11.6)	0.803^a^
Obstructive sleep apnea	12,194 (10.4)	143 (12.3)	0.041*^a^	930 (9.0)	16 (11.0)	0.404^a^
Arteriosclerosis	313 (0.3)	15 (1.3)	<0.001*^a^	13 (0.1)	0 (0)	0.669^a^
Cutaneous ulcer	137 (0.1)	74 (6.3)	<0.001*^a^	30 (0.3)	0 (0)	0.515^a^
Psychosis	944 (0.8)	2 (0.2)	0.015*^a^	44 (0.4)	0 (0)	0.430^a^
COPD	12,085 (10.3)	390 (33.4)	<0.001*^a^	1,241 (12.0)	76 (47.9)	<0.001*^a^
Traumatic head injury	626 (0.5)	2 (0.2)	0.089^a^	101 (1.0)	0 (0.0)	0.231^a^
Vasospasm	9 (0)	0 (0)	0.765^a^	0 (0)	0 (0)	N/A
Squamous cell carcinoma of the lung	0 (0)	63 (5.4)	<0.001*^a^	0 (0)	33 (22.6)	<0.001*^a^
Pneumonia	13,722 (11.7)	287 (24.6)	<0.001*^a^	2,100 (20.3)	81 (55.5)	<0.001*^a^
Large cell carcinoma of the lung	0 (0)	3 (0.3)	<0.001*^a^	0 (0)	0 (0)	N/A
Rheumatoid arthritis	1,498 (1.3)	17 (1.5)	0.592^a^	173 (1.7)	6 (4.1)	0.024*^a^
Ductal carcinoma of the breast	0 (0)	7 (0.6)	<0.001*^a^	0 (0)	(0)	N/A
Subclavian artery disease	15 (0)	0 (0)	0.699^a^	0 (0)	0 (0)	N/A
Teratoma	5 (0)	0 (0)	0.823^a^	0 (0)	0 (0)	N/A
Microangiopathy	2 (0)	0 (0)	0.888^a^	0 (0)	0 (0)	N/A
Down syndrome	152 (0.1)	0 (0)	0.218^a^	1	0	0.906^a^
Gastric erosion	4 (0)	0 (0)	0.842^a^	9 (0.1)	0 (0)	0.722^a^
Senile tremor	10 (0)	0 (0)	0.752^a^	0 (0)	0 (0)	N/A
Hispanic	2,411 (2.1)	0 (0)	<0.001*^a^	169 (1.6)	0 (0)	0.120^a^
Alcohol use				2,993 (29.0)	24 (16.4)	<0.001*^a^
Medication use No. (%)						
SSRI -present	37,354 (31.9)	304 (26.0)	<0.001*^a^	7,276 (70.2)	77 (52.7)	<0.001*^a^
CAI-present	28,844 (24.6)	261 (22.4)	0.073^a^	1,006 (9.7)	6 (4.1)	0.023*^a^
SGAP present	18,803 (16.1)	118 (10.1)	<0.001*^a^	1,685 (16.2)	30 (20.5)	0.163^a^
Memantine	18,900 (16.1)	184 (15.8)	0.729^a^	698 (6.7)	1 (0.7)	0.004*^a^
Buspirone	10,880 (9.3)	110 (9.4)	0.876^a^	1,185 (11.4)	2 (1.4)	<0.001*^a^
Valproate	17,864 (15.3)	173 (14.8)	0.682^a^	1,496 (14.4)	6 (4.1)	<0.001*^a^
Lab values: Mean ± SD						
Folate	11.7 **±** 4.2	11.7 **±** 3.8	0.992^b^	11.3 **±** 3.2	11.5 **±** 3.5	0.898^b^
B12	820.4 **±** 383.9	-	-	239 **±** 0	-	-
Vitamin D	28.9 **±** 15.3	32.0 **±** 14.5	0.454^b^	29.7 **±** 16.8	33.5 **±** 10.0	0.653^b^
TSH	3.2 **±** 10.6	2.6 **±** 3.9	0.472^b^	3.53 **±** 10.2	2.74 **±** 2.67	0.726^b^
Magnesium	2.0 **±** 0.3	1.9 **±** 0.3	0.030*^b^	1.924 **±** 0.4	1.8 **±** 0.3	0.064^b^
Serum calcium	9.1 **±** 0.7	9.0 **±** 0.8	0.068^b^	9.0 **±** 0.8	8.8 **±** 0.7	0.005*^b^
Homocysteine	15.0 **±** 13.1	9.0 **±** 8.3	0.517^b^	12.9 **±** 5.9	–	–

Results for continuous variables are presented as Mean ± SD, while discrete data are presented as percentage frequency. Pearson’s Chi-Square test is used to compare clinical risk factors and demographics in patients who have cancer vs. patients who do not have cancer. ^a^Pearson’s Chi-Squared test. ^b^Student’s *T-*test. *P-value < 0.05.

Among AD patients with metabolic encephalopathy but without cancer (AD + ME-C), older age was more common (*p* < 0.001). This group also presented with higher rates of anxiety, urinary tract infections, and alcohol use (*p* < 0.05). AD + C + ME patients were significantly more likely to present with hypertension, insomnia, cerebrovascular accidents, osteoporosis, secondary dementia, lung adenocarcinoma, small cell carcinoma of the lung, mild cognitive impairment, COPD, squamous cell carcinoma of the lung, pneumonia, and rheumatoid arthritis (all *p* < 0.05). AD+ME-C patients were more often treated with SSRIs, carbonic anhydrase inhibitors, memantine, buspirone, and valproate (*p* < 0.05) and had elevated serum calcium levels (*p* = 0.005).

### 3.2 Risk factors distinguishing cancer Alzheimer’s disease patients with and without metabolic encephalopathy (AD ± ME + C)

In all multivariable logistic regression models, the dependent variable was explicitly defined and coded as a binary outcome (1 = outcome of interest; 0 = reference group). Odds ratios (ORs) were interpreted consistently across all models such that OR > 1 indicates increased odds of the outcome group (coded as 1), whereas OR < 1 indicates increased odds of the reference group (coded as 0). The reference category for each model was explicitly defined in the corresponding table legend to ensure clarity and consistency of interpretation across analyses. The results of the adjusted analysis of the entire AD patient population are presented in Table 3. For Table 3 of outcome for presence of metabolic encephalopathy among cancer patients (AD + C + ME = 1; AD + C − ME = 0). OR > 1 indicates factors associated with AD + C + ME, while OR < 1 indicates factors associated with AD + C − ME (reference group). The Hosmer–Lemeshow test (*p* < 0.001; Cox and Snell *R*^2^ = 0.026) suggests a deviation from perfect calibration. However, given the large sample size, this test is highly sensitive and may detect minor, clinically negligible differences. Therefore, our calibration should be interpreted cautiously. Model performance was assessed using discrimination metrics (AUROC) and effect estimates. The area under the ROC curve (AUC = 0.656, 0.651–0.661) was used to assess model performance. Patients in the AD + C - ME group were characterized by chronic vascular, neurodegenerative, psychiatric, and treatment-related features. Dyslipidemia (OR 0.79, 95% CI 0.75–0.83), including a prior cerebrovascular accident (OR 0.89, 95% CI 0.83–0.96) and arteriosclerosis (OR 0.48, 95% CI 0.27–0.84), was significant (*p* < 0.05), indicating that stable vascular pathology was more common among patients without metabolic encephalopathy. Functional and neurologic characteristics including gait dysfunction (OR 0.71, 95% CI 0.62–0.82), mild cognitive impairment (OR 0.19, 95% CI 0.15–0.23), secondary dementia (OR 0.46, 95% CI 0.37–0.58), psychosis (OR 0.54, 95% CI 0.40–0.74), and nonspecific headache (OR 0.68, 95% CI 0.58–0.81) were also strongly associated with AD – ME + C, reflecting a chronic neurodegenerative phenotype rather than acute metabolic instability.

**Table 3:** Risk factors distinguishing Cancer Alzheimer’s disease patients with and without metabolic encephalopathy (AD + C ± ME).

Variables	*B-*value	Wald	Odds ratio	95% C.I.		*P*-value
				**Lower**	**Upper**	
Dyslipidemia	−0.239	83.6	0.787	0.748	0.829	<0.001*
Cerebrovascular accident	−0.114	9.7	0.893	0.831	0.959	<0.001*
Gait dysfunction	−0.340	23.3	0.712	0.622	0.815	<0.001*
Peripheral vascular disease	0.95	5.5	1.1	1.016	1.191	0.018*
Atrial fibrillation	0.95	6.6	1.1	1.024	1.182	0.010*
Anxiety	−0.152	17.9	0.859	0.800	0.922	<0.001*
Urinary tract infection	0.352	139.6	1.422	1.341	1.508	<0.001*
Upper respiratory tract infection	−0.492	32.2	0.612	0.516	0.725	<0.001*
Insulin use	−0.373	9.7	0.688	0.544	0.871	0.002*
Secondary dementia	−0.770	46.4	0.463	0.371	0.578	<0.001*
Cold sore	−2.6	13.1	0.076	0.019	0.307	<0.001*
Frailty	1.030	23.2	2.801	1.842	4.259	<0.001*
Advanced age	0.345	24.1	1.411	1.230	1.620	<0.001*
Mild cognitive impairment	−1.686	277.3	0.185	0.152	0.226	<0.001*
Headache unspecified	−0.386	19.2	0.680	0.575	0.808	<0.001*
Congestive heart failure	0.612	306.5	1.844	1.722	1.975	<0.001*
Obstructive sleep apnea	−0.122	11.2	0.885	0.824	0.950	<0.001*
Arteriosclerosis	−0.740	6.7	0.477	0.273	0.843	0.009*
Cutaneous ulcer	0.342	3.0	1.408	0.954	2.076	0.085
Psychosis	−0.610	15.2	0.544	0.400	0.738	<0.001*
COPD	0.086	7.0	1.090	1.023	1.161	0.008*
Traumatic head injury	0.644	33.8	1.903	1.532	2.365	<0.001*
Squamous cell carcinoma of the lung	1.795	64.8	6.019	3.888	9.318	<0.001*
Rheumatoid arthritis	0.265	10.5	1.303	1.110	1.530	<0.001*
Down syndrome	−2.324	5.343	0.021	0.014	0.702	<0.001*
Gastric erosion	3.088	25.6	21.935	6.630	72.571	<0.001*
Hispanic	0.145	3.2	0.865	0.738	1.015	0.075
SSRI present	−0.046	4.072	0.955	0.914	0.999	0.044*
CAI present	−0.930	639.0	0.394	0.367	0.424	<0.001*
Memantine	−0.558	162.0	0.572	0.525	0.624	<0.001*
Buspirone	0.270	65.1	1.310	1.227	1.399	<0.001*
Valproate	0.063	4.3	1.065	1.004	1.129	0.038*

Adjusted OR < 1 signifies factors that are associated with Cancer AD patients without metabolic encephalopathy (AD + C – ME), while OR>1 signifies factors that are associated with Cancer AD patients with metabolic encephalopathy (AD + C + ME). *Indicates statistical significance (*P* < 0.05) with a 95% confidence interval. Backward Stepwise model based on Likelihood Ratio was applied. Model assumptions were fulfilled. Multicollinearity and interactions among independent variables were checked and no significant interactions were found. Hosmer-Lemeshow test (*P* = <0.001), Cox & Snell (*R*^2^ = 0.026).

Several infectious and metabolic conditions were inversely associated with metabolic encephalopathy, including upper respiratory tract infection (OR 0.61, 95% CI 0.52–0.73), cold sores consistent with HSV reactivation (OR 0.08, 95% CI 0.02–0.31), insulin use (OR 0.69, 95% CI 0.54–0.87), and obstructive sleep apnea (OR 0.89, 95% CI 0.82–0.95). Importantly, Alzheimer’s disease–directed pharmacotherapy demonstrated strong protective associations: cholinesterase inhibitor use was associated with reduced odds of metabolic encephalopathy (OR 0.39, 95% CI 0.37–0.42), as was memantine therapy (OR 0.57, 95% CI 0.53–0.62). SSRI exposure also showed a modest association (OR 0.96, 95% CI 0.91–1.00). Memantine and cholinesterase inhibitor use were associated with lower odds of metabolic encephalopathy; however, these associations should be interpreted cautiously and may reflect underlying differences in patient characteristics rather than direct therapeutic effects.

In contrast, patients in the AD + ME + C group exhibited a profile associated with systemic illness, cardiovascular dysfunction, frailty, inflammation, and oncologic burden. Congestive heart failure emerged as a major cardiovascular predictor of metabolic encephalopathy (OR 1.84, 95% CI 1.72–1.98), accompanied by peripheral vascular disease (OR 1.10, 95% CI 1.02–1.19), atrial fibrillation (OR 1.10, 95% CI 1.02–1.18), and chronic obstructive pulmonary disease (OR 1.09, 95% CI 1.02–1.16). Frailty was one of the strongest predictors (OR 2.80, 95% CI 1.84–4.26), and advanced age significantly increased the odds of metabolic encephalopathy (OR 1.41, 95% CI 1.23–1.62), underscoring diminished physiologic reserve as a key driver of vulnerability.

Infectious and inflammatory triggers played a central role in AD + ME + C patients. Urinary tract infection was strongly associated with AD + ME + C (OR 1.42, 95% CI 1.34–1.51), consistent with its established role as a precipitant of delirium and encephalopathy in older adults. Traumatic head injury was associated with increased odds (OR 1.90, 95% CI 1.53–2.37), highlighting the additive impact of structural brain injury and metabolic stress. Oncologic and autoimmune conditions conferred particularly high risk: squamous cell carcinoma of the lung was associated with a six-fold increase in odds of metabolic encephalopathy (OR 6.02, 95% CI 3.89–9.32), rheumatoid arthritis increased risk by approximately 30% (OR 1.30, 95% CI 1.11–1.53), and severe gastrointestinal pathology such as gastric erosion showed the strongest association in the model (OR 21.94, 95% CI 6.63–72.57); however, given the magnitude of the odds ratio and potential for sparse data bias, this finding should be interpreted cautiously and may not reflect a precise estimate of effect. Psychotropic and neurologic medications were also associated with AD + ME + C, including buspirone (OR 1.31, 95% CI 1.23–1.40) and valproate (OR 1.07, 95% CI 1.00–1.13), likely reflecting treatment of behavioral disturbances, seizure risk, or neuropsychiatric instability in patients with advanced systemic illness. Collectively, these findings delineate two biologically trajectories: AD – ME + C, characterized by chronic neurodegenerative and vascular disease with preserved metabolic stability and higher use of cognitive-enhancing therapies, and AD + ME + C, defined by acute systemic insults, cardiovascular compromise, frailty, infection, malignancy, and inflammatory stress.

### 3.3 Risk factors associated with AD patients without metabolic encephalopathy with and without cancer (AD – ME ± C)

Table 4 summarizes the multivariable logistic regression analysis identifying clinical factors associated with Alzheimer’s disease patients AD – ME ± C. In Table 4 of the outcome for the presence of cancer among AD patients without metabolic encephalopathy (AD – ME + C = 1; AD – ME − C = 0), OR > 1 indicates factors associated with AD – ME + C. OR < 1 indicates factors associated with AD – ME − C (reference group). The Hosmer–Lemeshow test (*p* < 0.001; Cox and Snell *R*^2^ = 0.014) suggests a deviation from perfect calibration. Model performance was primarily assessed using discrimination metrics (AUROC) and effect estimates. The area under the ROC curve (AUC = 0.732, 0.717–0.746) was used to assess model fitness.

**Table 4:** Risk factors associated with AD patients without metabolic encephalopathy with and without cancer (AD-ME ± C).

Variables	B value	Wald	Odds ratio	95% C.I.		*P*-value
				Lower	Upper	
Insomnia	−0.470	9.921	0.625	0.457	0.837	0.002*
Dyslipidemia	0.353	28.119	1.423	1.249	1.622	<0.001*
Gait dysfunction	−0.342	3.198	0.710	0.488	1.033	0.074
Peripheral Vascular Disease	0.402	17.896	1.489	1.241	1.801	<0.001*
Urinary Tract Infection	−0.599	25.562	0.550	0.436	0.693	<0.001*
Upper Respiratory Tract Infection	−0.823	7.248	0.439	0.241	0.799	0.007*
Insulin Use	−2.139	16.83	0.118	0.042	0.327	<0.001*
Secondary Dementia	−1.030	7.326	0.357	0.169	0.753	0.007*
Advanced Age	−1.626	10.483	0.197	0.074	0.526	<0.001*
Mild Cognitive Impairment	−0.249	2.736	0.780	0.581	1.047	<0.098
Congestive Heart Failure	0.589	38.766	1.801	1.497	2.1688	<0.001*
Arteriosclerosis	1.669	37.266	5.309	3.106	9.074	<0.001*
Cutaneous ulcer	3.959	493.997	52.427	36.976	74.334	<0.001*
Psychosis	−1.308	3.404	0.270	0.067	1.085	0.065
COPD	1.150	271.229	3.157	2.754	3.620	<0.001*
Traumatic Head Injury	−1.079	2.303	0.340	0.084	1.370	0.129
SSRI Present	−0.265	14.038	0.768	0.668	0.881	<0.001*

Adjusted OR < 1 denotes factors that are associated with AD-ME-C, while OR > 1 denotes factors that are associated with AD – ME + C. *Indicates statistical significance (*P* < 0.05) with a 95% confidence interval. Classification table (overall correctly classified percentage = 73.2%) and area under the ROC curve (AUC = 0.732, 0.717–0.746) were applied to check model fitness. Backward Stepwise model based on Likelihood Ratio was applied. Model assumptions were fulfilled. Multicollinearity and interactions among independent variables were checked and no significant interactions were found. Hosmer-Lemeshow test (*P* = <0.001), Cox and Snell (*R*^2^ = 0.014).

Several cardiovascular and systemic comorbidities were significantly associated with AD – ME ± C. Dyslipidemia was independently associated with increased odds of cancer coexistence (OR = 1.42, 95% CI 1.25–1.62, *p* < 0.001), as were peripheral vascular disease (OR = 1.49, 95% CI 1.24–1.80, *p* < 0.001) and congestive heart failure (OR = 1.80, 95% CI 1.50–2.17, *p* < 0.001), underscoring the contribution of systemic vascular pathology in this subgroup. Arteriosclerosis demonstrated a particularly strong association (OR = 5.31, 95% CI 3.11–9.07, *p* < 0.001), suggesting that advanced atherosclerotic disease is a key determinant of cancer coexistence in AD patients without metabolic encephalopathy. Pulmonary disease and tissue integrity markers further distinguished AD – ME ± C. Chronic obstructive pulmonary disease (COPD) was associated with a more than threefold increase in the odds of AD – ME + C (OR = 3.16, 95% CI 2.75–3.62, *p* < 0.001). Most notably, the presence of cutaneous ulcers was associated with markedly increased odds of cancer coexistence (OR = 52.43, 95% CI 36.98–74.33, *p* < 0.001); however, this large effect size should be interpreted cautiously, as it may reflect sparse data bias or model instability.

In contrast, several factors were significantly associated with AD – ME - C. Advanced age was strongly associated with lower odds of cancer coexistence (OR = 0.20, 95% CI 0.07–0.53, *p* < 0.001), consistent with competing risks and survivorship effects. Insomnia (OR = 0.63, 95% CI 0.46–0.84, *p* = 0.002), urinary tract infection (OR = 0.55, 95% CI 0.44–0.69, *p* < 0.001), and upper respiratory tract infection (OR = 0.44, 95% CI 0.24–0.80, *p* = 0.007) were also preferentially associated with AD-ME-C, suggesting that infectious and sleep-related disturbances are more prominent in non-oncologic AD populations.

Metabolic and neuropsychiatric factors further differentiated the groups. Insulin use was strongly associated with AD – ME – C (OR = 0.12, 95% CI 0.04–0.33, *p* < 0.001), as was secondary dementia (OR = 0.36, 95% CI 0.17–0.75, *p* = 0.007). Selective serotonin reuptake inhibitor (SSRI) use was also significantly associated with lower odds of cancer coexistence (OR = 0.77, 95% CI 0.67–0.88, *p* < 0.001). Other factors, including gait dysfunction, mild cognitive impairment, psychosis, traumatic head injury, and upper-level neuropsychiatric diagnoses, showed trends but did not reach statistical significance.

Collectively, these results indicate that AD patients without metabolic encephalopathy who have coexisting cancer are characterized by a distinct profile associated with vascular disease, cardiopulmonary comorbidity, dyslipidemia, and markers of systemic illness, whereas AD patients without metabolic encephalopathy and cancer more commonly exhibit advanced age, infectious complications, metabolic treatment exposure, and neuropsychiatric management. This divergence suggests different risk factors among AD – ME ± C patients, with important implications for risk stratification and management.

### 3.4 Risk factors associated with AD patients with metabolic encephalopathy with and without cancer (AD + ME ± C)

Table 5 presents the results of a multivariable logistic regression model identifying clinical factors associated with Alzheimer’s disease patients with metabolic encephalopathy with and without coexisting cancer (AD + ME ± C). In Table 5 of the outcome for the presence of cancer among AD patients with metabolic encephalopathy (AD + ME + C = 1; AD + ME − C = 0). OR > 1 indicates factors associated with AD + ME + C. OR < 1 indicates factors associated with AD + ME − C (reference group).

**Table 5:** Risk factors associated with AD patients with metabolic encephalopathy with and without cancer (AD + ME ± C).

Variables	B value	Wald	Odds ratio	95% C.I.		*P*-value
				Lower	Upper	
Cerebrovascular accident	1.245	26.264	3.471	2.157	5.588	<0.001
Osteoporosis	−0.735	3.357	0.480	0.219	1.058	0.067
Anxiety	−0.782	3.061	0.458	1.191	1.099	0.080
Secondary Dementia	2.291	16.404	9.889	3.263	29.975	<0.001*
Mild Cognitive Impairment	1.648	11.223	5.197	1.981	13.269	<0.001*
COPD	2.036	106.247	7.664	5.203	11.288	<0.001*
SSRI Present	1.189	34.715	3.272	2.210	4.874	<0.001*
Memamtine	−2.102	4.285	0.122	0.017	0.894	0.038*
Busprione	−1.735	5.826	0.166	0.043	0.722	0.016*
Valproate	−1.424	10.244	0.241	0.101	0.576	0.001*

Adjusted OR < 1 signifies factors that are associated with AD + ME – C while OR > 1 signifies factors that are associated with AD + ME + C. *Indicates statistical significance (*P* < 0.05) with a 95% confidence interval. Classification table (overall correctly classified percentage = 87.3%) and area under the ROC curve (AUC = 0.873, 0.842–0.905) were applied to check model fitness. Backward Stepwise model based on Likelihood Ratio was applied. Model assumptions were fulfilled. Multicollinearity and interactions among independent variables were checked and no significant interactions were found. Hosmer-Lemeshow test (*P* = <0.017), Cox and Snell (*R*^2^ = 0.046).

The Hosmer–Lemeshow test (*p* < 0.017; Cox and Snell *R*^2^ = 0.046) suggests a deviation from perfect calibration. Model performance was assessed using discrimination metrics (AUROC) and effect estimates. Area under the ROC curve (AUC = 0.873, 0.842–0.905) was applied to check model fitness. Several factors were strongly and independently associated with AD + ME + C. A history of cerebrovascular accident was associated with more than a threefold increase in the odds of cancer coexistence (OR = 3.47, 95% CI 2.16–5.59, *p* < 0.001), suggesting a substantial contribution of vascular burden in this subgroup. Secondary dementia showed the strongest association with AD + ME + C, conferring nearly a tenfold increase in odds (OR = 9.89, 95% CI 3.26–29.98, *p* < 0.001), highlighting the importance of non-primary neurodegenerative risk factors in cancer-associated encephalopathy in AD patients. Similarly, mild cognitive impairment was significantly associated with AD + ME + C (OR = 5.20, 95% CI 1.98–13.27, *p* < 0.001), suggesting that patients earlier in the cognitive disease continuum may be particularly vulnerable to oncologic and metabolic stressors.

Pulmonary disease also emerged as a prominent risk factor. Chronic obstructive pulmonary disease (COPD) was associated with a markedly association with AD + ME + C (OR = 7.66, 95% CI 5.20–11.29, *p* < 0.001), consistent with shared inflammatory, hypoxic, and smoking-related pathways. In addition, selective serotonin reuptake inhibitor (SSRI) use was independently associated with AD + ME + C (OR = 3.27, 95% CI 2.21–4.87, *p* < 0.001), potentially reflecting higher psychiatric burden, greater symptom complexity, or differential prescribing patterns in cancer-affected patients.

**Figure 2:**
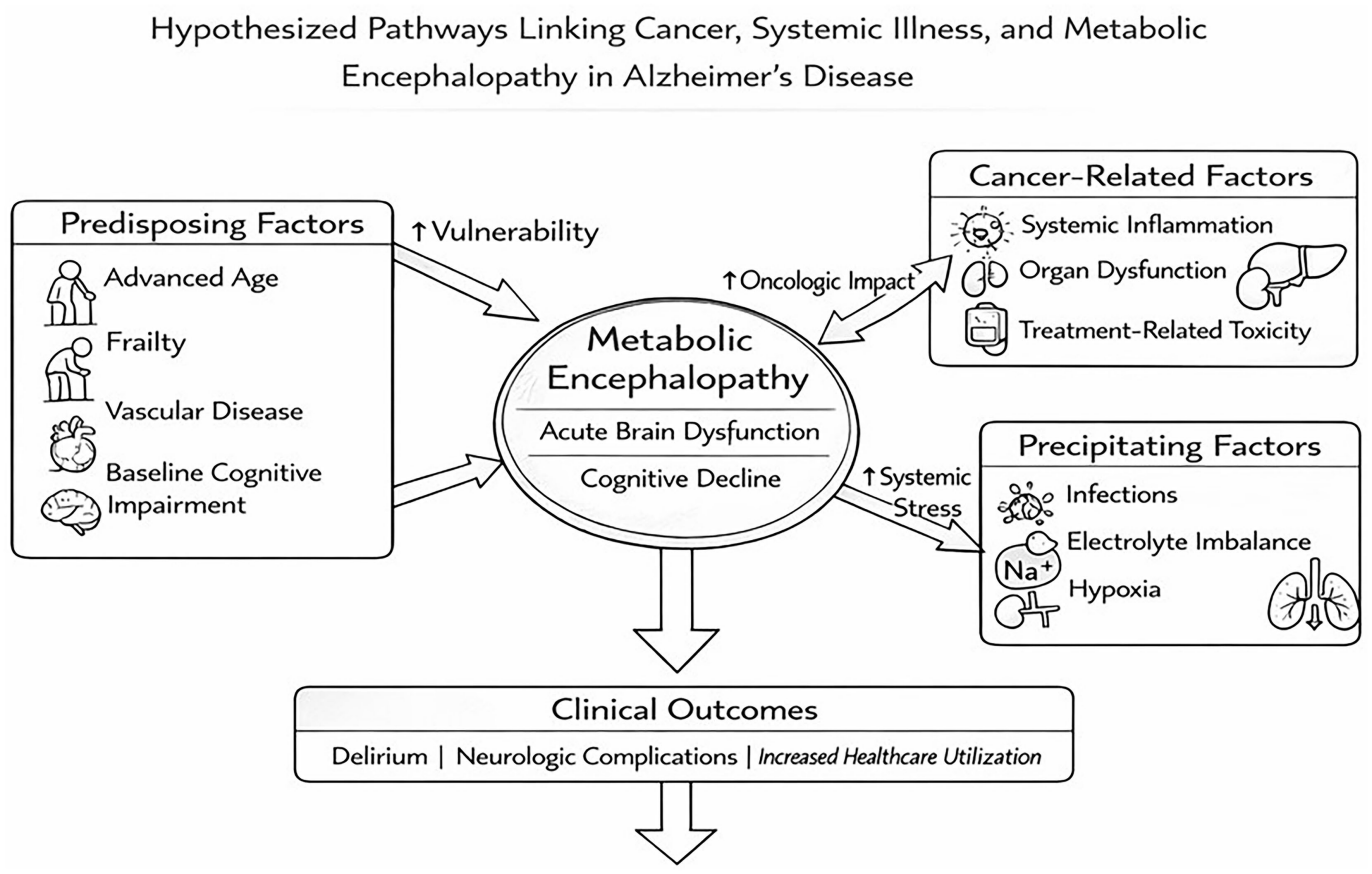
Hypothesized pathways linking cancer, systemic illness, and metabolic encephalopathy in Alzheimer’s disease. Predisposing factors (age, frailty, vascular disease, baseline cognitive impairment) increase baseline vulnerability. Cancer-related processes (systemic inflammation, organ dysfunction, treatment-related toxicity) contribute to chronic metabolic stress. Acute precipitating factors (infection, electrolyte imbalance, hypoxia) act as proximal triggers. The interaction of these domains may contribute to the development of metabolic encephalopathy, representing acute brain dysfunction superimposed on underlying neurodegeneration.

In contrast, several factors were preferentially associated with AD + ME - C. Use of memantine was associated with significantly lower odds of cancer coexistence (OR = 0.12, 95% CI 0.02–0.89, *p* = 0.038), as was treatment with buspirone (OR = 0.17, 95% CI 0.04–0.72, *p* = 0.016) and valproate (OR = 0.24, 95% CI 0.10–0.58, *p* = 0.001). In the age-stratified analyses (e.g., <75 years, ≥75 years) to assess effect modification by age. The overall pattern of associations remained consistent across strata, although effect sizes were generally stronger in older patients, consistent with increased physiologic vulnerability. Figure 2 illustrates a multifactorial conceptual framework describing the development of metabolic encephalopathy (ME) in patients with Alzheimer’s disease (AD), particularly in the context of cancer. ME is conceptualized as an acute manifestation of systemic and neurologic vulnerability, arising from the interaction of baseline susceptibility, chronic systemic stressors, and acute physiologic insults. At the foundation of this framework are predisposing factors, including advanced age, frailty, cardiovascular and cerebrovascular disease, and baseline cognitive impairment due to AD. These factors reduce physiologic and neurocognitive reserve, creating a state of heightened vulnerability and lowering the threshold for acute brain dysfunction. Superimposed on this baseline state are cancer-related factors, which act as chronic systemic stressors. These include systemic inflammation, immune dysregulation, organ dysfunction (e.g., hepatic, renal, or pulmonary impairment), and treatment-related toxicity from oncologic therapies. Together, these processes contribute to metabolic instability, oxidative stress, and impaired homeostatic regulation, further sensitizing the brain to injury.

Acute precipitating factors serve as proximal triggers that directly induce encephalopathy. These include infections (e.g., urinary tract infections, pneumonia), electrolyte imbalances (e.g., sodium or calcium disturbances), and hypoxia. Such insults disrupt cerebral metabolism, neurotransmitter balance, and neuronal signaling, leading to rapid deterioration in cognitive function. The convergence of these three domains baseline vulnerability, chronic systemic stress, and acute triggers culminates in metabolic encephalopathy, characterized by acute global brain dysfunction and exacerbation of underlying neurodegenerative processes. Clinically, ME reflects a state of reduced physiologic resilience and increased systemic burden, rather than a single causal pathway. Overall, this framework highlights ME as a syndromic endpoint of interacting pathophysiologic processes, providing a mechanistic basis for understanding the heterogeneity and clinical complexity observed in AD patients with cancer.

## 4 Discussion

This study examined clinical factors associated with Alzheimer’s disease (AD) patients with and without metabolic encephalopathy (ME), stratified by cancer status. Our results suggest that AD patients who also had ME tended to have a greater burden of systemic illness and physiological stress. In contrast, cancer-associated AD patients without ME more closely resembled a chronic disease profile. These observations suggest that ME is associated with a subgroup with greater clinical complexity and vulnerability, consistent with prior literature linking multimorbidity to acute cognitive decompensation.

### 4.1 Risk factors associated with AD + ME + C versus AD + ME − C

Among AD patients with metabolic encephalopathy, the presence of cancer was strongly associated with vascular, pulmonary, neurocognitive, and psychiatric factors, suggesting that cancer-related metabolic encephalopathy is associated with heightened systemic vulnerability rather than isolated oncologic burden (Holmes et al., 2024). A prior cerebrovascular accident was associated with more than a threefold increase in the odds of cancer coexistence, highlighting the convergence of vascular brain injury, impaired cerebral reserve, and systemic illness in this subgroup. Cerebrovascular disease has been consistently linked to both cancer risk and delirium susceptibility, through shared mechanisms involving endothelial dysfunction, chronic inflammation, and impaired blood–brain barrier integrity (Libby et al., 2019; Iadecola, 2013).

We observed that secondary dementia demonstrated the strongest association with AD + ME + C patients, underscoring the importance of non-primary neurodegenerative etiologies such as vascular dementia, medication-related cognitive impairment, or metabolic and inflammatory brain injury. These conditions may contribute to greater vulnerability, possibly promoting encephalopathy (Inouye et al., 2014; Fong et al., 2015; Desai et al., 2026). Interestingly, mild cognitive impairment was also strongly associated with AD + ME + C, suggesting that patients earlier in the cognitive disease continuum may be particularly susceptible to oncologic, inflammatory, and metabolic insults. This aligns with evidence that patients with preserved baseline cognition experience more pronounced delirium and encephalopathy under systemic stressors, as their cognitive decline may relate more to acute metabolic dysregulation than to chronic neuronal loss (Cunningham and Maclullich, 2013).

Pulmonary disease emerged as a major risk factor in AD + ME + C patients. For example, chronic obstructive pulmonary disease (COPD) was associated with AD + ME + C patients. COPD contributes to chronic hypoxemia, systemic inflammation, and oxidative stress, factors that independently increase cancer risk and predispose to metabolic encephalopathy through impaired cerebral oxygen delivery and mitochondrial dysfunction (Wilson et al., 2020). The association between SSRI use and AD + ME + C likely reflects greater psychiatric burden, associated with greater medical complexity and clinical instability, rather than a direct pharmacologic effect. Depression and anxiety are common in cancer patients and have been independently linked to delirium, encephalopathy, and adverse neurologic outcomes, particularly in the context of polypharmacy and systemic inflammation (Fardell et al., 2023; Breitbart et al., 2015; Nathaniel et al., 2026).

In contrast, AD + ME - C were more likely to be treated with memantine, buspirone, and valproate. These findings suggest a different clinical profile among AD + ME – C patients characterized by greater emphasis on neuropsychiatric stabilization and cognitive symptom management, potentially reflecting lower systemic inflammatory burden and reduced oncologic stress. Memantine’s NMDA-receptor antagonism may also confer partial protection against excitotoxic injury during metabolic stress, while mood-stabilizing agents may reduce agitation-associated metabolic demand (Ojo et al., 2025; Maldonado, 2018; Gainey et al., 2024).

### 4.2 Risk factors associated with AD – ME + C versus AD – ME − C

In AD patients without encephalopathy, advanced vascular disease, systemic frailty, and pulmonary pathology were associated with cancer (AD – ME + C), suggesting a pattern dominated by chronic systemic illness rather than acute neurologic destabilization. Dyslipidemia, peripheral vascular disease, and congestive heart failure were all independently associated with AD – ME + C patients, emphasizing the central role of cardiometabolic and vascular pathology. These conditions promote chronic inflammation, oxidative stress, and immune dysregulation, biologic pathways that are strongly implicated in carcinogenesis and cancer progression (Hanahan and Weinberg, 2011; Ridker et al., 2017; Akinwumi et al., 2025).

Arteriosclerosis had a strong association, indicating that advanced atherosclerotic disease may signal long-standing systemic injury and reduced physiologic reserve. Importantly, despite this heavy vascular burden, these patients did not present with metabolic encephalopathy. This is consistent with the idea that encephalopathy reflects acute decompensation rather than cumulative disease severity alone (Kondo et al., 2022). Pulmonary disease was again significant: COPD was associated with more than a threefold increase in the odds of cancer in AD – ME + C patients, consistent with its well-known links to lung and extrapulmonary malignancies via smoking-related, inflammatory, and hypoxic pathways (Durham and Adcock, 2015; Brewer et al., 2025; Broughton et al., 2025).

Another notable finding was the strong association between cutaneous ulcers and the coexistence of cancer in the AD – ME – C group. Cutaneous ulcers likely reflect severe systemic illness, chronic inflammation, impaired wound healing, malnutrition, and advanced frailty; conditions commonly seen in advanced malignancy (Maida et al., 2008; Babalola et al., 2025; O’Brien et al., 2025). The relative absence of this feature in AD + ME + patients suggests that tissue breakdown and chronic systemic decline are more characteristic of cancer-associated AD without encephalopathy. We again observed that memantine, buspirone, and valproate use were more frequent in AD + ME – C patients, reinforcing that those receiving structured neuropsychiatric management tended to have a less oncologically and systemically burdened profile.

Taken together, these findings reveal two different profiles among cancer-associated AD patients. AD + ME + C patients are characterized by acute systemic stress, pulmonary disease, cerebrovascular injury, psychiatric complexity, and early-stage cognitive vulnerability, whereas AD – ME + C patients exhibit a pattern dominated by chronic vascular disease, frailty, tissue breakdown, and long-standing systemic illness. Metabolic encephalopathy was associated with greater systemic and neurologic vulnerability in cancer-associated AD. These distinctions have important implications for risk stratification and early identification of reversible precipitants, but causal links cannot be established from this study.

The observed associations between neurocognitive/psychotropic medications (e.g., memantine, cholinesterase inhibitors, SSRIs) and clinical outcomes should be interpreted with caution. These are susceptible to confounding by indication (patients on such therapies may have better care, closer monitoring, or less severe illness) and may reflect underlying disease stage or healthcare engagement rather than direct therapeutic effects. Therefore, these findings do not imply causal benefit of the medications.

## 5 Conclusion

This study identifies different clinical profiles among Alzheimer’s disease patients based on the presence of metabolic encephalopathy and cancer. Metabolic encephalopathy was associated with patterns of increased systemic vulnerability including vascular, pulmonary, and neuropsychiatric comorbidities, whereas patients without metabolic encephalopathy had profiles more consistent with chronic disease burden. These findings highlight the heterogeneity of AD populations and underscore the importance of recognizing metabolic encephalopathy as a marker of clinical complexity. Future studies incorporating longitudinal data and more detailed clinical measures are needed to clarify temporal relationships and validate these associations.

## 6 Limitations

Several limitations should be considered when interpreting these findings. The retrospective and observational nature of the analysis precludes causal inference, and the identified associations may reflect residual confounding despite multivariable adjustment. The reliance on diagnostic coding may introduce misclassification bias, particularly for metabolic encephalopathy, cancer subtype, and neuropsychiatric conditions, which can vary in severity and clinical interpretation. The detailed information on cancer stage, treatment modality, tumor burden, and timing relative to encephalopathy onset was unavailable, limiting the ability to assess dose–response relationships or treatment-related effects.

The medication associations may reflect confounding by indication, as drugs such as memantine, valproate, buspirone, and SSRIs are prescribed in response to symptom severity and clinical context rather than randomly assigned. Measures of frailty, inflammation, nutritional status, and metabolic derangement were inferred from clinical diagnoses rather than direct physiologic or laboratory data. Also although model discrimination was high, the modest explained variance suggests that unmeasured factors, including social determinants of health, hospitalization severity, and acute physiologic parameters, likely contribute to the development of metabolic encephalopathy in this population. Although discrimination metrics (AUROC and classification accuracy) suggested acceptable model performance, additional calibration-focused measures such as Brier scores and calibration plots were not performed and represent an important area for future methodological refinement. Moreover, internal validation (e.g., bootstrapping or cross-validation) was not performed. Future studies will incorporate resampling-based internal validation and class imbalance–adjusted performance metrics, including balanced accuracy and precision–recall analysis, to improve the robustness and clinical applicability of predictive modeling in heterogeneous dementia populations.

Several statistical limitations should be considered. The Hosmer–Lemeshow test indicated statistically significant deviation from perfect calibration in some models; however, this test is highly sensitive in large datasets and may over-detect minor departures from ideal fit. Therefore, these findings should be interpreted cautiously, and additional calibration measures (e.g., calibration plots or Brier scores) would strengthen future analyses. Also model discrimination was modest in certain analyses (AUC ~ 0.65), limiting predictive performance. The very large odds ratios observed for some variables may reflect sparse data bias or quasi-separation, resulting in unstable and potentially inflated effect estimates. These findings should therefore be interpreted cautiously, particularly in subgroup analyses with limited event counts. Future studies using penalized regression methods or sensitivity analyses (e.g., exclusion of rare variables) are needed to confirm the robustness of these associations.

Given the cross-sectional design, all findings should be interpreted as associations, and no causal or temporal inferences can be drawn from this study. Mortality outcomes and time-to-event data were not available in the registry, precluding survival analysis. As a result, the study cannot directly assess the impact of metabolic encephalopathy on mortality, and conclusions are limited to associations with clinical characteristics rather than survival outcomes.

Despite these limitations, the large sample size and consistent effect estimates across multiple biologically plausible domains strengthen the validity of the findings. Future prospective studies incorporating granular clinical, laboratory, and oncologic data are needed to clarify causal pathways, refine risk stratification, and identify targeted interventions to prevent metabolic encephalopathy in vulnerable Alzheimer’s disease patients with cancer.

## Data Availability

The raw data supporting the conclusions of this article will be made available by the authors, without undue reservation.

## Glossary

AD: Alzheimer’s disease
ADIA: Alzheimer’s disease with irritability and anger
HTN: Hypertension
CVA: Cerebrovascular accident
PVD: Peripheral vascular disease
AF: Atrial fibrillation
UTI: Urinary tract infection
URI: Upper respiratory infection
GI: Gastrointestinal
MCI: Mild cognitive impairment
CHF: Congestive heart failure
OSA: Obstructive sleep apnea
COPD: Chronic obstructive pulmonary disease
RA: Rheumatoid arthritis
SSRIs: Selective serotonin reuptake inhibitors
ChEIs: Cholinesterase inhibitors
SGAs: Second-Generation antipsychotics
SPSS: Statistical package for social sciences
SD: Standard deviation
AUROC: Area under the receiver operating characteristic curve
NIH: National Institute of Health
